# Lymphocytic Choriomeningitis Virus Infections and Seroprevalence, Southern Iraq

**DOI:** 10.3201/eid2612.201792

**Published:** 2020-12

**Authors:** Hussein Alburkat, Anne J. Jääskeläinen, Ali M. Barakat, Hassan J. Hasony, Tarja Sironen, Haider Al-hello, Teemu Smura, Olli Vapalahti

**Affiliations:** University of Helsinki, Helsinki, Finland (H. Alburkat, A.J. Jääskeläinen, T. Sironen, T. Smura, O. Vapalahti);; Helsinki University Hospital, Helsinki (A.J. Jääskeläinen, O. Vapalahti);; University of Basrah, Basrah, Iraq (A.M. Barakat, H.J. Hasony);; Finnish Institute for Health and Welfare, Helsinki (H. Al-hello)

**Keywords:** Lymphocytic choriomeningitis virus, immunofluorescence assay, seroprevalence, IgM, IgG, mammarenavirus, zoonoses, viruses, Iraq

## Abstract

Acute febrile neurological infection cases in southern Iraq (N = 212) were screened for lymphocytic choriomeningitis virus (LCMV). Two LCMV IgM–positive serum samples and 2 cerebrospinal fluid samples with phylogenetically distinct LCMV strains were found. The overall LCMV seroprevalence was 8.8%. LCMV infections are common and associated with acute neurological disease in Iraq.

Lymphocytic choriomeningitis virus (LCMV) is a rodentborne pathogen that belongs to the genus *Mammarenavirus*, family *Arenaviridae*. The house mouse (*Mus musculus*) is considered the reservoir of LCMV (*1*). Humans can be infected with LCMV by inhaling particles contaminated with rodent excreta, during organ transplantation, or congenitally during pregnancy (*2*). The symptoms of LCMV infection range from subclinical to severe (*3*); severe infections may manifest as meningitis or encephalitis or as a congenital syndrome including microcephaly, for example (*4*).

Because of the cosmopolitan distribution of its reservoir host, LCMV most likely circulates globally. However, most epidemiologic studies on LCMV have been conducted in Europe, the United States, Japan, and China (*5*–*10*). The presence and seroprevalence of LCMV infections in the Middle East region have remained unknown (*11*,*12*). We report on LCMV seroprevalence, acute LCMV infections, and characterization of phylogenetically distinct local LCMV strains in southern Iraq.

## The Study

**Figure 1:**
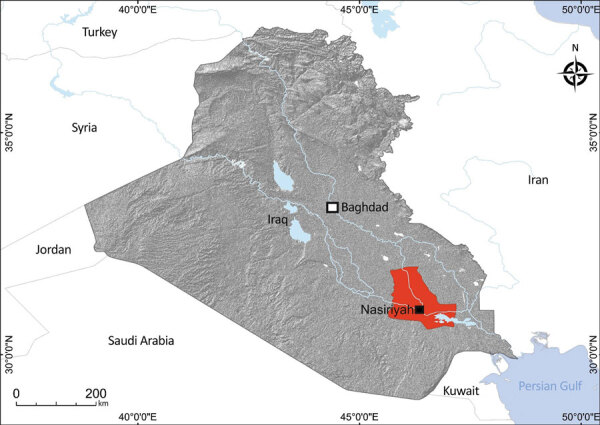
Study site (red) in Dhi Qar Governorate, Nasiriyah region, Iraq, from where serum and cerebrospinal fluid samples were collected from persons in rural and urban areas and screened for lymphocytic choriomeningitis virus.

We collected 261 serum samples (from 171 acute febrile patients and 90 healthy controls) in Nasiriyah region, Dhi Qar governorate, southern Iraq (Figure 1) during 2012–2016. In addition, we collected 41 cerebrospinal fluid (CSF) samples from another set of acute febrile patients. All samples were stored at −70°C.

We studied the occurrence of LCMV infection in the Nasiriyah region of southern Iraq by screening 171 serum and 41 CSF samples, from patients with fever and neurologic manifestations, for LCMV RNA and IgM and IgG. The inclusion criteria for the study were acute febrile illness and neurologic symptoms such as headache, muscle weakness, or fatigue (Table 1). The mean duration of illness was 4.29 days (range 3–7 days). We used the IgG positivity in serum samples from the symptomatic patients as well as healthy controls to estimate the LCMV seroprevalence in the region. Ethics permissions were obtained and stored in the Al Hussain General Teaching Hospital and Bint Al Huda Maternity and Children Teaching Hospital in the Nasiriyah region, southern Iraq.

**Table 1:** Signs and symptoms observed among 212 patients with acute febrile illness and neurologic symptoms screened for lymphocytic choriomeningitis virus, southern Iraq

Sign or symptom	Percentage
Fever	100
Headache	90
Joint pain	68
Vertigo	61
Severe malaise	48
Chills	46
Cough	46
Abdominal pain	34
Drowsiness	30
Anorexia	28
Stiff neck	28
Nausea	21
Retroorbital pain	19
Diarrhea	18
Vomiting	10
Confusion	8
Severe muscle weakness	6
Conjunctivitis	3
Lymphadenopathy	3
Rash	2
Ataxia	1
Shortness of breath	1

We extracted viral RNA from acute infection samples (serum and CSF) (140 μL/sample) using a QIAamp Viral RNA Mini kit (QIAGEN, https://www.qiagen.com) according to the manufacturer’s instructions. We performed a pan-arena reverse transcription PCR (RT-PCR) using SuperScript II One-Step RT-PCR system with Platinum Taq High Fidelity (Invitrogen, https://www.thermofisher.com), and primers described previously (*13*). RT-PCR products (»300–400 bp) were sequenced using the Sanger method; sequencing was performed by the Sequencing laboratory of Institute for Molecular Medicine Finland FIMM Technology Centre, University of Helsinki. For antibody detection, indirect LCMV IgM and IgG immunofluorescence assays (IFAs) were conducted, as described previously (*6*). In general, IFAs are not very specific assays; therefore, one could assume cross-reaction between LCMV and other mammarenaviruses. The specificity and sensitivity of IFA were not examined in this study.

The serum samples derived from patients with fever and neurologic symptoms were screened by IFA for both LCMV IgM and IgG. LCMV IgM was found in 2 serum samples (2/171) derived from patients with acute febrile illness; both serum samples were negative for LCMV IgG and LCMV RNA. These patients (a 65-year-old woman and a 70-year-old man) had fever and neurologic symptoms (Table 2).

**Table 2:** Clinical observations in 4 patients with test results positive for lymphocytic choriomeningitis virus, southern Iraq*

Observation	CSF RNA–positive patients			IgM–positive patients	
	Male. no. 11	Female. no. 64		Male. no. 61	Female. no. 38
Diagnosis	Meningoencephalitis	Meningitis		None	No diagnosis
Duration of illness	7	4		3	3
Symptoms	Fever	Fever		Fever	Fever
	Chills	Chills		Headache	Chills
	Headache	Headache		Drowsiness	Headache
	Cough	Cough		Vertigo	General malaise
	Retroorbital pain	Retroorbital pain		Joint pain	Vertigo
	Severe muscle weakness	Severe malaise			Abdominal pain
	Drowsiness	Drowsiness			Fatigue
	Vertigo	Vertigo			
	Joint/ bone pain	Joint pain			
	Stiff neck				

*CSF, cerebrospinal fluid; LCMV, lymphocytic choriomeningitis virus.

**Figure 2:**
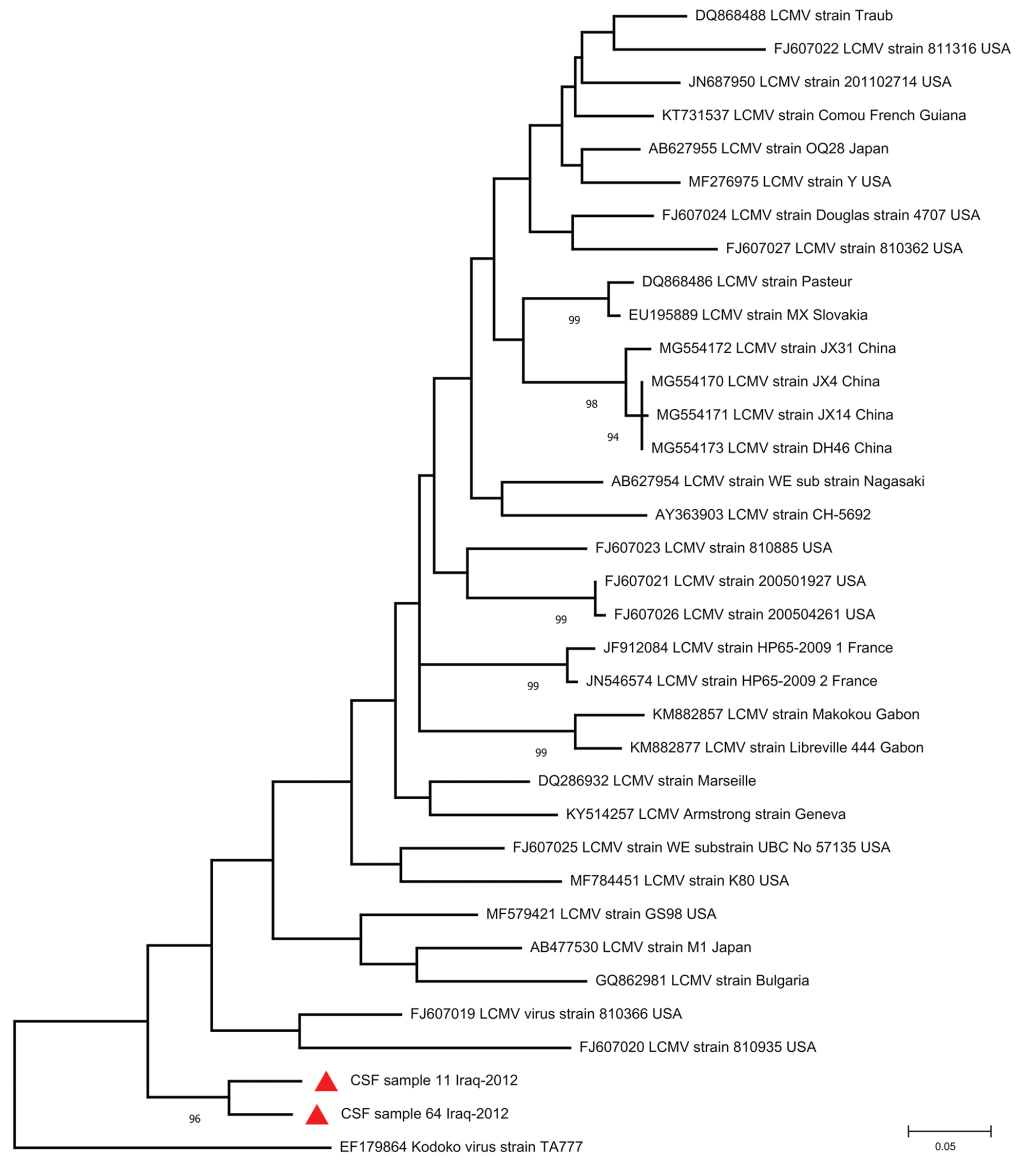
Phylogenetic tree of lymphocytic choriomeningitis virus strains detected in southern Iraq (red triangles) and reference sequences. GenBank accession number, strain name, and country of origin are indicated for reference sequences. Bootstrap support values >70 are shown at the nodes. The phylogenetic tree was constructed using MEGA version 7 (https://www.megasoftware.net) and the maximum-likelihood algorithm on the basis of partial large segments of Kodoko virus and partial large segment sequences corresponding to sites 3210–3604 of strain Armstrong (accession no. NC_004291). Scale bar indicates substitutions per nucleic acid site. CSF, cerebrospinal fluid.

Two CSF samples (from a 35-year-old woman and a 50-year-old man) derived from patients with fever and neurologic symptoms (Table 2) were positive for LCMV RNA by using panarenavirus RT-PCR and sequencing. Phylogenetic analysis showed that both of the sequences grouped with other LCMV strains but formed a distinct subcluster (Figure 2). No corresponding serum samples were available for these patients, but CSF samples were further tested for LCMV IgM and IgG; all were negative.

The overall LCMV IgG seroprevalence was 8.8% (23/261) in all serum samples. The seroprevalence of LCMV in our study was 12.2% (11/90) in the healthy control group and 7% (12/171) in the acute febrile patients. This difference was not statistically significant (p = 0.2 by χ^2^ test). Because the patient samples were collected early after onset of illness (3–7 days), IgG had not yet developed; IgG serostatus thus reflects past immunity in this patient group. The healthy control population (mean age 42.9 years) was younger than acute febrile patients (mean age 46.3 years). Healthy men (7.9%) were more often LCMV seropositive than were women (5.6%), but in patients with acute febrile illness, the gender ratio was reversed (3.9% in women, 2.8% in men).The detected LCMV IgG–positive samples were derived from all age groups (21–80 years of age) included in this study. The differences concerning residency, age, and gender were not statistically significant. IgG titers measured among positive samples ranged from 20 to 80 in IFA.

## Conclusions

Only limited information is available on LCMV infections beyond the United States, Europe, Japan, and China. In this work, we focused on both acute febrile infections (presence of IgM antibodies in serum or LCMV RNA in CSF) and seroprevalence of LCMV in southern Iraq. Considerable LCMV seroprevalence was detected in the Nasiriyah region of southern Iraq, and acute LCMV infection was confirmed by demonstration of LCMV RNA in 2 CSF samples and IgM antibodies in 2 serum samples. The phylogenetic analyses of these 2 findings revealed that the new sequences formed a unique subcluster, ancestral to previously known LCMV strains.

Overall, the seroprevalence rate (8.8%) of LCMV infection characterized in this study is in line with seroprevalences detected earlier in many countries in Europe, in which it varies from 5.0% in Finland (*14*) to 36% in a special subset in a rural area of the northern Croatian island of Vir (*15*). Collectively, the seroprevalence and detection of acute infection, including 2 phylogenetically distinct sequences, provide evidence that LCMV circulates in southern Iraq, and it is causing infections leading to acute neurologic manifestations in the population. More sequence data are needed to extend the knowledge on the molecular epidemiology and evolution of LCMV. In addition, further research to characterize LCMV in rodent reservoirs in southern Iraq is needed to plan vector control and public health recommendations.
