# Correction: Vol. 26, No. 12

**DOI:** 10.3201/eid2706.C12706

**Published:** 2021-06

**Keywords:** errata, erratum, correction

GenBank accession numbers have been added for the sequenced viral sequences from Lymphocytic Choriomeningitis Virus Infections and Seroprevalence, Southern Iraq (H. Alburkatet al.). The article has been corrected online (https://wwwnc.cdc.gov/eid/article/26/12/20-1792_article).

